# Pigmented anatomy in Carboniferous cyclostomes and the evolution of the vertebrate eye

**DOI:** 10.1098/rspb.2016.1151

**Published:** 2016-08-17

**Authors:** Sarah E. Gabbott, Philip C. J. Donoghue, Robert S. Sansom, Jakob Vinther, Andrei Dolocan, Mark A. Purnell

**Affiliations:** 1 Department of Geology, University of Leicester, Leicester LE1 7RH, UK; 2 Department of Earth Sciences, University of Bristol, Bristol BS8 1RJ, UK; 3 Faculty of Life Sciences, University of Manchester, Manchester M20 6RT, UK; 4 Texas Materials Institute, The University of Texas at Austin, Austin, TX 78712, USA

**Keywords:** cyclostomes, Mazon Creek, melanosomes, retinal pigment epithelium

## Abstract

The success of vertebrates is linked to the evolution of a camera-style eye and sophisticated visual system. In the absence of useful data from fossils, scenarios for evolutionary assembly of the vertebrate eye have been based necessarily on evidence from development, molecular genetics and comparative anatomy in living vertebrates. Unfortunately, steps in the transition from a light-sensitive ‘eye spot’ in invertebrate chordates to an image-forming camera-style eye in jawed vertebrates are constrained only by hagfish and lampreys (cyclostomes), which are interpreted to reflect either an intermediate or degenerate condition. Here, we report—based on evidence of size, shape, preservation mode and localized occurrence—the presence of melanosomes (pigment-bearing organelles) in fossil cyclostome eyes. Time of flight secondary ion mass spectrometry analyses reveal secondary ions with a relative intensity characteristic of melanin as revealed through principal components analyses. Our data support the hypotheses that extant hagfish eyes are degenerate, not rudimentary, that cyclostomes are monophyletic, and that the ancestral vertebrate had a functional visual system. We also demonstrate integument pigmentation in fossil lampreys, opening up the exciting possibility of investigating colour patterning in Palaeozoic vertebrates. The examples we report add to the record of melanosome preservation in Carboniferous fossils and attest to surprising durability of melanosomes and biomolecular melanin.

## 1. Introduction

Lampreys and hagfish are the only living jawless vertebrates; they occupy crucial intermediate phylogenetic positions between the nearest invertebrate relatives of vertebrates—urochordates and cephalochordates—and gnathostomes. The condition of hagfish eyes has proved particularly influential in scenarios of eye evolution. In contrast to lampreys, which possess a sophisticated eye with a lens, iris and eye muscles, hagfish eyes lack such structures and, unlike almost all other vertebrates, including lampreys, the retinal epithelium of hagfish is devoid of pigment granules [1]. This condition has been interpreted to reflect a rudimentary intermediate evolutionary grade in the gradual assembly of the vertebrate eye [2,3]. However, extant cyclostomes cannot be taken as accurate proxies for their last common ancestor [4]. Hagfish and lampreys differ significantly in their morphology, and like all living representatives of deep-branching clades, they have acquired, lost and transformed characteristics compared with their last common ancestor [4]. Consequently, the hypothesis that hagfish eyes reflect an evolutionary intermediate of invertebrate eyespots and vertebrate camera eyes, or a faithful vestige of such an ancestral state [3], is open to question. Fossil evidence of the condition of eyes in ancient cyclostomes has the potential to resolve this important issue in understanding the evolution of the vertebrate eye.

Melanosomes are the organelles that manufacture and store the pigment melanin; typically, they are spherical to elliptical bodies 0.5–2 µm in diameter. Their shape and distribution in fossils, especially in bird feathers [5,6] and non-avian dinosaurs [7], have been used to reconstruct colour and colour-based patterns, providing insight into the ecology of extinct vertebrates. Most previous work has focused on tetrapod vertebrates, and the existence and significance of melanosomes in older vertebrates, which sit outside the gnathostome (jawed vertebrate) crown group, are confined to a single study on the eyes of *Tullimonstrum gregarium* from the Mazon Creek, allowing the affinity of this previously enigmatic organism to be resolved [8]. In part, this bias towards tetrapod vertebrates reflects the fact that dark stains in basal vertebrates have been assumed to be decay-resistant tissues, such as cartilage, with the identity of eyes, for example, being based on the position of paired stains in the head. Interpreting dark stains merely to indicate that eyes were present, however, tells us nothing about their sensory sophistication and thus provides scant evidence for understanding the origins and evolution of sensory systems in basal vertebrates.

## 2. Results

### (a) Scanning electron microscopic analysis

Scanning electron microscopy (SEM) analysis of *Mayomyzon pieckoensis* has revealed some aspects of morphology not previously described in detail or recognized, including an oral disc, fin radials and otic capsule containing statoliths. These are described and figured in detail (see the electronic supplementary material, ‘Details on the anatomy of *Mayomyzon*’ and figures S1 and S2). Here, we focus on anatomical features preserved as dark stains and their details, resolved through SEM imaging.

Both *M. pieckoensis* and *Myxinikela siroka* preserve large, paired, dark circular to oval structures in the head, and comparative anatomical analysis provides unequivocal evidence that these structures are eyes [9,10]. Diverse Mazon Creek vertebrates, including members of Chondrichthyes, Acanthodes and Actinopterygii, preserve similar paired, circular, dark structures in the head, and in these taxa hard-tissue anatomical landmarks allow confident interpretation that such structures are eyes. The only other possibility—that they are otic capsules—can be discounted because otic capsules are also preserved, just posterior of the dark paired circles in three specimens of *Mayomyzon* (see the electronic supplementary material, figure S2)*.* These otic capsules are preserved in pyrite and contain apatite statoliths, similar to the arrangement of eyes and otic capsules in the Mazon Creek elasmobranch *Bandringa* [11].

SEM imaging and energy-dispersive X-ray analysis reveal that the eyes and other anatomical features that occur as dark stains in both *Mayomyzon* and *Myxinikela* are preserved as carbon-rich films. The films are composed of masses of oblate to cylindrical bodies, and/or exhibit amorphous textures that are frequently cracked (see the electronic supplementary material, figure S3). The eyes are of the former type.

**Figure 1.**
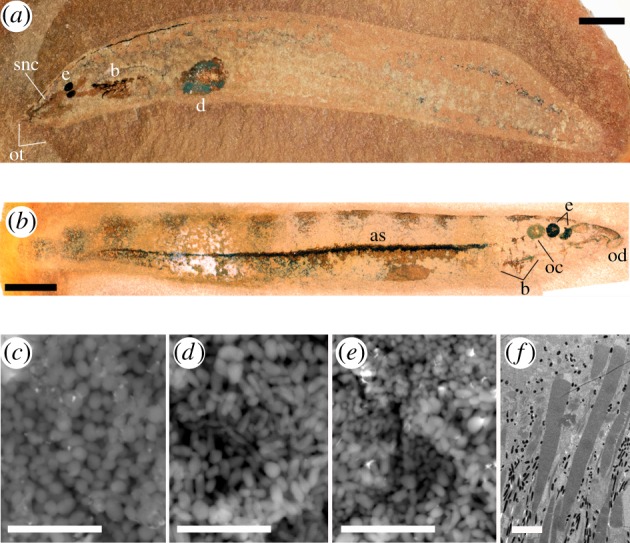
Fossil cyclostomes from the Mazon Creek Lagerstätte. (*a*) *Myxinikela siroka* (PF15373)*.* Scale bar, 5 mm. (*b*) *Mayomyzon pieckoensis* (ROMV56800b) showing clearly defined pigmented stripes along the dorsal surface. Scale bar, 5 mm. (*c*–*e*) Back-scattered electron (BSE) SEM images of melanosomes present in the eyes of (*c*) *Myxinikela siroka* (PF15373), (*d*) *Mayomyzon pieckoensis* (LEIUG 123268) and (*e*) *Mayomyzon pieckoensis* (ROM56806); note two distinct melanosome morphologies, which is typical of the RPE of fish [12–14]. All scale bars, 5 µm. Not all material preserved as carbon within the fossils shows these textures; for example, in *Mayomyzon* the oral disc and pharynx comprise sheet-like carbon with associated pyrite. Ellipsoid/oblate textures are not evident in carbon patches beyond the margins of the body in any of the taxa studied. (*f*) Radial TEM image of the retina of an extant fish (*Rhinogobius*). Dark pigment granules (melanosomes) are elliptical in the base of the image and spherical at the top of the image. Decay-induced collapse of the RPE would result in a fossilized structure with both elliptical and oblate melanosome morphologies. Scale bar, 5 µm. Image courtesy of Gengo Tanaka. e, eye; ot, oral tentacles; snc, forked subnasal cartilage; b, branchial structure; d, digestive organ; oc, otic capsule, od, oral disc; as, axial structure. (Online version in colour.)

In the eyes of *Mayomyzon*, the bodies are 0.8–1.2 µm long and 0.38–0.47 µm wide, with rounded termini; in three specimens (LEIUG123268, ROM47555 and ROM56806) the bodies occur as a smaller, more oblate morphotype and a larger, more cylindrical morphotype (figure 1*e*). In *Myxinikela*, the bodies in the eyes are generally oblate in shape, but of varying sizes, between 0.6–1.15 µm long and 0.45–0.77 µm wide (figure 1*c*). Four specimens of *Mayomyzon* also preserve in the centre of each eye a circular structure in relief (380 µm × 500 µm across in the holotype PF5687), which we interpret as the lens. These structures are similar to those seen in the Mazon Creek elasmobranch *Bandringa* where an ‘unpigmented zone’ in the eye is interpreted to be the lens [11].

**Figure 2.**
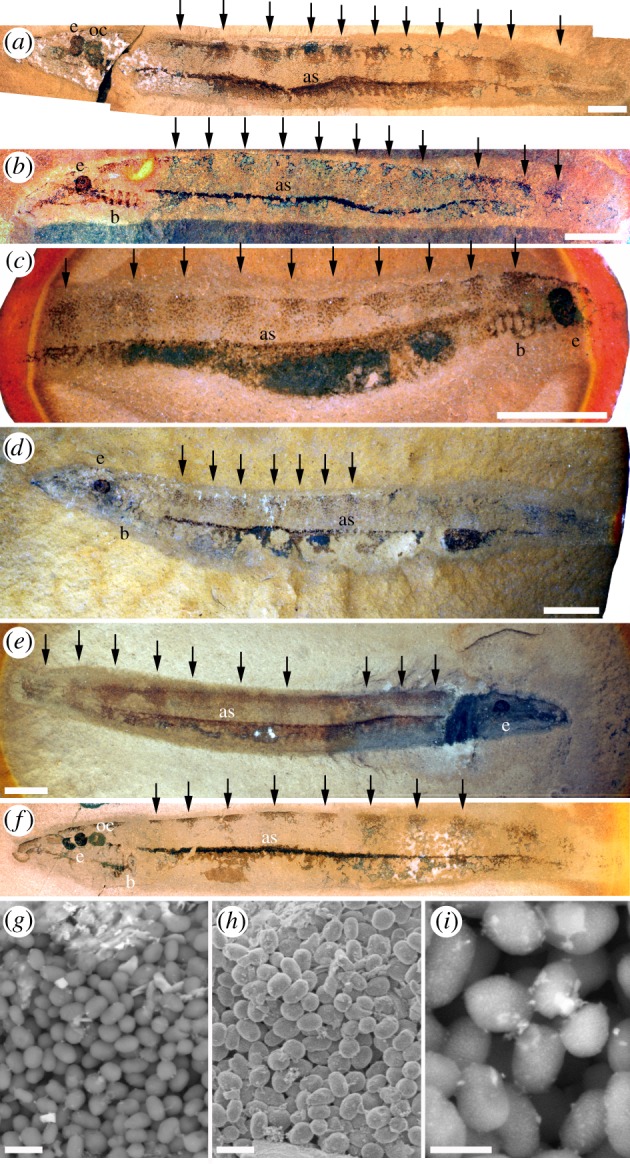
Bars of colour patterning along the dorsal surface on *Mayomyzon pieckoensis.* The dorsoventrally orientated ‘stripes’ (black arrows) comprise carbon of amorphous and elliptical microbody textures. (*a*) Specimen ROMV56787, (*b*) specimen PF5687a, (*c*) specimen ROMV56828a, (*d*) specimen PF5539, (*e*) specimen ROMV56788b and (*f*) specimen ROMV56800b. (*a*–*f*) Scale bars, 5 mm. (*g–i*) SEM images of melanosomes from the stripes in specimen ROMV56800b demonstrating relatively uniform size and shape of microbodies. (*g*) BSE image of melanosomes. Scale bar, 1 µm. (*h*) Secondary Electron image of melansomes. Scale bar, 1 µm. (*i*) BSE image showing that melanosomes are composed of many small melanin granules. Scale bar, 500 nm. e, eye; b, branchial structure; oc, otic capsule, as, axial structure. See also the electronic supplementary material, figure S1. (Online version in colour.)

Branchial structures in *Mayomyzon* and *Myxinikela* are composed of carbon in both sheet-like and oblate microbody masses. In addition, in *Mayomyzon*, broad, regularly spaced, dorsoventrally oriented dark-coloured bars along the flanks of the trunk and the dark axial line comprise oblate microbodies typically 0.7 µm long and 0.5 µm wide (figures 1*b* and 2; electronic supplementary material, figure S2*d*). Microbodies associated with the branchial structures, flank bars and axial line are more uniform in their size and shape than those seen in the eyes, and, unlike the eyes, highly cylindrical forms are absent.

### (b) Time of flight secondary ion mass spectrometry analysis

**Figure 3.**
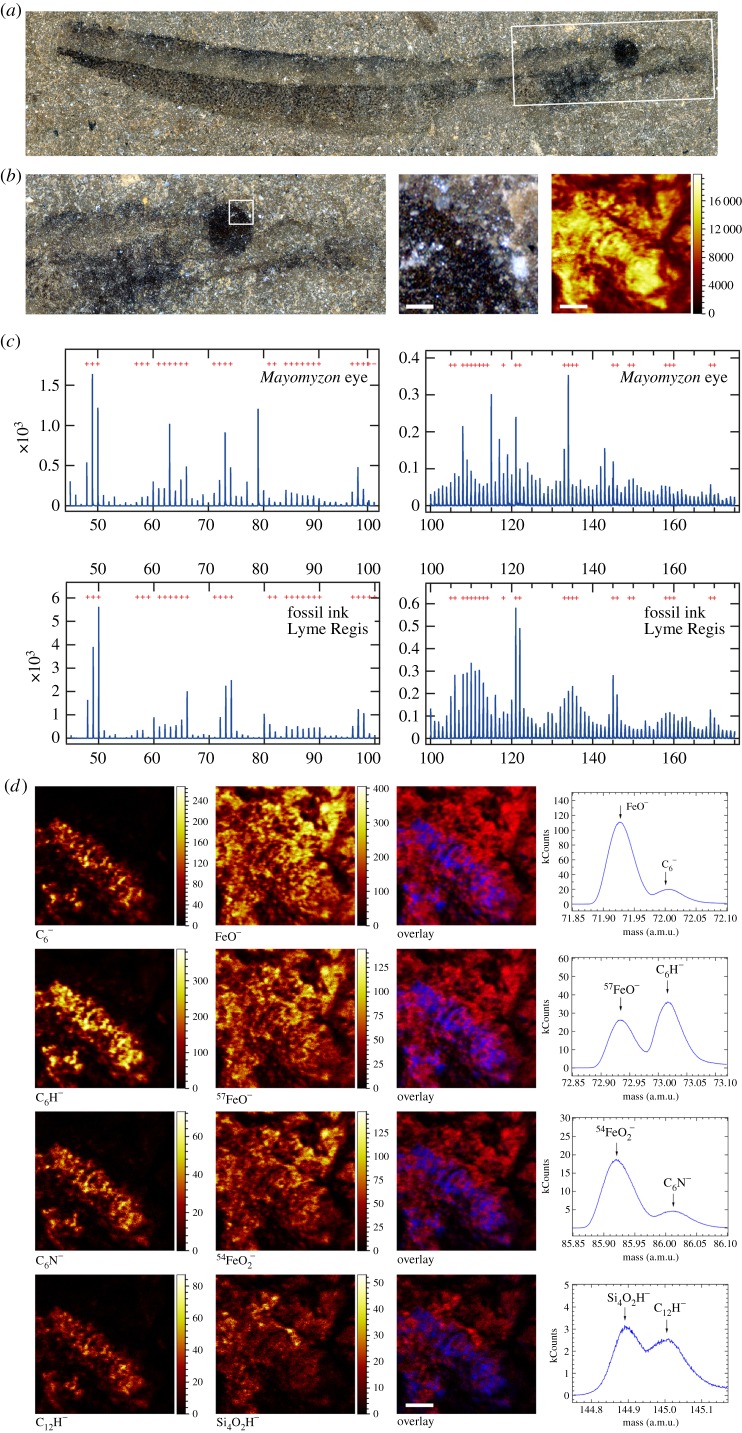
TOF-SIMS analysis of melanosomes in the eye of *Mayomyzon* LEIUG123268. (*a*) Complete specimen of *Mayomyzon* LEIUG123268. (*b*) Optical overview of head and region analysed under the TOF-SIMS (optical view and total yield; scale bars, 100 µm). (*c*) Comparison of TOF-SIMS spectrum from the eye in comparison with a fossil melanin reference (Jurassic cephalopod ink). (*d*) Representative examples of spatial distribution of secondary ion fragments previously assigned to fossil melanin [15]: 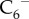, C_6_H^−^, C_6_N^−^ and C_12_H^−^, together with matrix-related fragments: FeO^−^, ^57^FeO^−^, 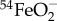 and Si_4_O_2_H^−^. Scale bar, 100 µm. See also the electronic supplementary material, figures S4 and S6 for additional maps and spectra. (Online version in colour.)

In order to determine the composition of the microbodies, we analysed those in the eye and the bars on the dorsal surface of the trunk of *Mayomyzon* using time of flight secondary ion mass spectrometry (TOF-SIMS). The secondary ion spectra acquired (figure 3; electronic supplementary material, figure S4) contain all the organic secondary ion fragments that characterize fossil melanin, and these are distinctly expressed in the eye (figure 3*d*; electronic supplementary material, figure S4) [12,16]. SEM observations show that the layers of microbodies occur in between cement and sediment grains, and thus the spectra derived from the eye represent the TOF-SIMS signal of both the microbodies and the matrix of the concretion (figure 3*d*). In order to disentangle these, we mapped a location including the eye and some of the adjacent matrix (figure 3). For example, figure 3*d* shows four melanin-specific fragments (in this case, 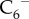) clearly localized to the eye and not the matrix; the same is true for the other characteristic secondary ions (electronic supplementary material, figure S4). The inorganic ions are expressed throughout. This reveals that the melanin and the matrix-related fragments can be mass separated.

Comparing the spectrum of melanin characteristic fragments from a selected region of interest defined by the TOF-SIMS map directly with a reference spectrum acquired from a pure melanin sample reveals that the fossil eye displays all the peaks characteristic of melanin (red crosses, figure 3*c*). However, it does not precisely match its pure eumelanin counterpart, because inorganic ions in the matrix and fossil overprint the subdued melanin spectrum. Thus, detailed visual comparison of the overall spectra of the melanosomes within the fossil eye to melanin references is difficult. Consequently, rather than rely on subjective visual inspection, we used principal components analysis to conduct a multivariate statistical comparison of the relative intensity distribution of the melanin-specific peaks from the eye of *Mayomyzon* (LEIUG123268) and from fresh, artificially matured and fossil melanin samples (including amphibians, birds, mammals, fish and cephalopods) from a wide range of localities with distinct diagenetic histories [15], and from non-melanin samples. In this analysis (electronic supplementary material, figure S5), spectra from *Mayomyzon* plot among other fossil melanin samples and are distinct from non-melanin controls, including sediment adjacent to the lamprey eye shown in figure 3. We have furthermore extracted the melanin characteristic ions from the eye and the adjacent sediment (electronic supplementary material, figure S6) in order to illustrate the relative contribution to the principal components analysis.

## 3. Discussion

### (a) Interpretation of the microbodies

Interpretation of the microbodies rests on testing two alternative hypotheses: either they are microbial remains or they are fossil melanosomes. Using a variety of evidence, we can reject the former hypothesis and interpret the microbodies as melanosomes.

In terms of their consistent morphology and limited size range, the microbodies we describe closely resemble melanosomes in fishes [12,13] and amphibians [17]. Whether size and shape of microbodies is a reliable diagnostic feature to distinguish between fossil melanosomes and fossil bacteria is debated [18–20], but arguments that melanosomes and bacteria overlap in size, shape and preservation potential [21,22] are challenged by data demonstrating otherwise [18,23]. Bacteria stated to be identical in shape and size to melanosomes [19] are too large for any known extant or fossil melanosome [15,18] and generally exhibit much more diverse consortia, morphologically and taxonomically, than can be compared to melanosome associations.

The strongest evidence that the microbodies are melanosomes comes from their localization to tissues known from comparative anatomical analysis to be pigmented in life, and their exclusion from non-pigmented tissues. This is precisely the distribution of microbodies that is predicted by the hypothesis that they are melanosomes, but interpreting them as fossil bacteria relies on a non-parsimonious argument that bacteria and/or their preservation have localized only to pigmented tissues. Furthermore, there is an additional level of anatomical localization of microbody morphology in *Mayomyzon*: eyes have two distinct morphotypes, whereas the dorsal bars, brachial structures and axial line are homogeneous. Detailed examination of the eyes of three specimens reveals microbodies of distinct cylindrical and oblate morphology, the two morphologies often seen to be segregated into distinct areas (figure 1*e*). This is the configuration of melanosomes in the vertebrate retinal pigmented epithelium (RPE): more cylindrically shaped melanosomes occur in the apical part, and melanosomes of oblate shape in the basal layer (see below). An identical arrangement of microbodies to those in *Mayomyzon* occurs in the eyes of osteichthyan fish taxa from the Mazon Creek, including *Elonichthys peltigerus*, *Platysomus circularis* and *Esconicthys apopyris* [8]. In other Lagerstätte, heterogeneous microbodies confined to fossil fish eyes are also interpreted as melanosomes of the RPE [12,13,21].

Conversely, in *Mayomyzon* the dorsal bars, brachial structures and axial line microbodies are uniformly oblate and show very little size variation. All of these features can be related to pigmented anatomy in life. In many extant lampreys, the branchial structures are heavily pigmented, especially in ammocoetes (the branchial pigmentation patterns of which are used to differentiate species [24]). The dark, regularly spaced dorsal bars in *Mayomyzon*, composed exclusively of oblate microbodies, are interpreted as integument pigment patterning. No extant lamprey shows such vertical bars, but other pigmentation patterns, notably countershading and mottling, are common [25], and larvae (ammocoetes) of some species possess vertical stripes of pigmentation on their dorsal surfaces [24]. That the dark axial line is the gut [9] is not inconsistent with an interpretation of melanosomes in places along its length. Numerous species of fish possess a melanosome-rich peritoneal mesothelium that envelops the alimentary tract, and rarely fish form a melanized layer within the intestinal wall [26]. Another possibility is that this structure is an expression of the lateral line, the neuromasts of which are known to be highly pigmented in many extant lamprey taxa [25].

Chemical evidence, provided by TOF-SIMS analyses, shows that spectra from *Mayomyzon* and other melanin samples are directly comparable. The distinct relative intensity of these peaks, which provide a fingerprint signature for melanin, is seen in the eye and not in the surrounding sediment (electronic supplementary material, figure S4). Multivariate analysis was carried out using principal components analysis to compare the relative intensity distribution of the melanin-specific peaks originating from fresh, artificially matured, fossil melanin and non-melanin samples (controls) [15]. The data from *Mayomyzon* plot among samples of fossil melanin; crucially they plot away from controls (including the sedimentary rock in which the fossil is preserved), providing independent evidence that microbodies are composed of melanin and are thus melanosomes (electronic supplementary material, figure S5).

### (b) Skin pigmentation patterning in *Mayomyzon*

The regularly spaced bars along the dorsal surface of the trunk in *Mayomyzon* are interpreted as evidence of trunk melanophores, preserving the original skin pigmentation pattern and allowing a rare opportunity to reconstruct aspects of skin pigmentation patterns in a Palaeozoic vertebrate. Colour patterning has a diverse range of functions, from camouflage through to overt sexual display, as well as thermoregulation in ectotherms [16]. The presence of countershading and vertical striping is strong evidence for *Mayomyzon* living in clear waters in which there is a strong light gradient between the surface the bottom. Studies of modern European perch show a distinct correlation in the presence of vertical striping and degree of countershading to the turbidity of the lake they are found in [27]. Furthermore, vertical striping is usually associated with shallow-water fishes in which stripes may serve in outline breaking against heterogeneous backgrounds with a vertical component, such as roots, reeds or corals [28].

### (c) The pigmented eyes of *Mayomyzon* and *Myxinikela*

In vertebrate eyes, the iris, choroid and RPE all contain melanosomes. The dark eyes of the fossil cyclostomes may contain melanosomes from any of these tissues, which may be superimposed upon decay-induced collapse of the eye. But significantly, only the RPE contains heterogeneous melanosomes of different sizes and shapes [12,14], as seen in three *Mayomyzon*. In the vertebrate RPE cylindrical and oblate/spherical forms are separated into layers [13], again as we see in *Mayomyzon*.

However, an unequivocal RPE is seen in only three of nine *Mayomyzon* specimens studied. Most *Mayomyzon* and *Myxinikela* preserve eyes composed of oblate melanosomes, which vary in size with just a few cylindrical forms. This suggests that the split through the specimen, creating part and counterpart, is critical in revealing the layered, dual melanosome structure of the RPE. While the choroid and iris also contain melanosomes, they are known to be very homogeneous in both size and shape in extant lamprey [29]. Thus, there are two hypotheses to explain the oblate melanosome eyes in *Mayomyzon* and *Myxinikela*: either they contain RPE that is not exposed at the surface of the fossil, or they lack RPE. Given the clear evidence of dual melanosome RPE in three specimens, the former hypothesis is the more parsimonious.

Pigments also occur in association with light-sensing organs in invertebrate chordates, but we can reject the hypothesis that the pigmented structures in *Myxinikela* and *Mayomyzon* are similar to the light-detecting ‘eye’ of non-vertebrate chordates because they are paired, comparatively large and comprise a mass of melanosomes of dual morphology characteristic of vertebrates [8]. *Mayomyzon* also preserves evidence of a lens. This contrasts with the simple ‘eye’ in amphioxus, which comprises a single pigment cup with a few pigment cells, and the ocellus of larval tunicates, which comprises a single large cell containing pigment [2,3].

### (d) Phylogenetic placement of *Mayomyzon* and *Myxinikela* using new data

The evolutionary significance of our observations is contingent on the phylogenetic affinity of *Myxinikela* and *Mayomyzon*, and indeed the interrelationships of the living hagfishes, lampreys and jawed vertebrates. Support for a cyclostome clade of hagfishes and lampreys [4] is now overwhelming, based on phylogenetic analyses of protein-coding genes (e.g. [30–32]), the shared presence of cyclostome-specific non-coding microRNA families and demonstration that the traditional support from morphology for a lamprey–gnathostome clade is mostly based on out-moded data [4]. By contrast, the affinity of the fossil taxa has not been the subject of much scrutiny since the original description of *Mayomyzon* as a lamprey [9] and *Myxinikela* as a hagfish [10]. To this end, we corrected and revised an existing cladistic matrix [33] in the light of new anatomical observations, also using taphonomic data based on decay experiments on lamprey and hagfish [34,35] (electronic supplementary material, ‘Phylogenetics’). Phylogenetic analyses using both parsimony and Bayesian inference confirm that *Myxinikela* is a total-group hagfish and that *Mayomyzon* is a total-group lamprey. Importantly, in *Myxinikela*, the presence of hagfish synapomophies, including the oral tentacle cartilages and the forked subnasal cartilage, discount the possibility that this fossil has undergone significant stemward slippage [34,36].

### (e) Hagfish eyes are degenerate, not primitively simple

In the context of cyclostome monophyly, the presence of pigmented eye tissue, most probably RPE, in *Myxinikela* indicates that the common ancestor of hagfishes and lampreys possessed pigmented eyes and that this aspect of eye anatomy in extant hagfish has been simplified through degeneracy, rather than reflecting a primitively simple condition. Though in and of itself this is but one aspect of hagfish eye anatomy, the biology of the RPE in vertebrates suggests that this belies a broader degeneracy of the hagfish eye, including the lens and neural retina. This is because the RPE is multifunctional in vertebrate eyes, particularly in its promotion of the development and maintenance of the photoreceptor cell layer, as well as its role in reducing glare by absorbing excess light [37]. Thus, the eyes of extant hagfish are best interpreted to reflect degeneracy from a more complex eye, comparable with the eyes of extant lampreys and jawed vertebrates.

No extant chordates possess organs that are intermediate between the light-sensitive ‘eye spots’ of non-vertebrate cephalochordates and ascidians, and the image-forming camera-style eye of lampreys and gnathostomes. Under the scenario that hagfish are basal (i.e. sister group to lamprey + gnathostomes), their simple, unpigmented eye anatomy could be taken as a useful proxy for the condition of the early vertebrate eye and used to elucidate the sequence of events that took place in early vertebrate eye evolution [2]. This is more difficult under the hypothesis of cyclostome monophyly (hagfish + lamprey = Cyclostomata), but it has been postulated that the hagfish eye reflects a failure in eye development to proceed beyond that of an earlier stage in vertebrate eye evolution, echoing the plesiomorphic condition for the vertebrate eye and potentially shedding light on the anatomy of an even earlier evolutionary stage in eye development [3]. Our data indicate that the eyes of extant hagfishes are degenerate and are not an appropriate model for the evolutionary assembly of the vertebrate eye.

## 4. Summary

The discovery of melanosomes in Mazon Creek cyclostomes provides information on both ecological and evolutionary aspects of these animals deep in their history.

Until recently, the fossil record has remained mute on aspects of vertebrate eye evolution, simply because it did not provide sufficiently well-preserved eyes with adequate resolution to be informative. As such only data from extant taxa could be brought to bear on reconstructing the series of evolutionary steps that led from a primitive ‘eye spot’ through to image formation in the vertebrate camera-style eye [2,3]. However, the recent discovery of fossil fish eyes with ultrastructural fidelity of preservation has shown that, albeit rarely, the fossil record may capture detailed aspects of eye anatomy [13]. Our new cladistic analyses confirm that *Myxinikela* is a hagfish, and the recognition of pigmented eyes in this taxon indicates that the eyes of extant hagfish are degenerate, not primitively simple. This parallels data from embryology showing that hagfish have lost, rather than primitively lacked, vertebrate characters [38]. Our fossil evidence suggests that the last common ancestor of vertebrates had an eye that was at least as complex as that of lampreys.

## 5. Material and methods

### (a) Material

Fossil cyclostomes are from the Mazon Creek Lagerstätte (Francis Creek Shale Member, Late Carboniferous; approx. 307 Ma). They are from pit 11, which constitutes part of the shallow marine Essex fauna. Specimens occur as well-preserved but largely two-dimensional dark brown/black and green/brown remains within siderite concretions (smallest concretion 40 mm long and 30 mm wide; largest 94 mm long and 65 mm wide). Occasionally white kaolinite occurs on the surface of the fossil but this is of secondary diagenetic origin. Nine specimens of the lamprey *Mayomyzon pieckoensis* (LEIUG123268, ROMV56806, ROMV56788a, ROM56828b, ROMV56800a,b, PF10788a,b, PF5687, PF5688, PF15382) and the only known specimen of the hagfish *M. siroka* (PF15373a,b) were studied. Specimens are housed in the collections of the University of Leicester (LEIUG), Field Museum, Chicago (PF), and Royal Ontario Museum, Toronto (ROM). None of the specimens could be analysed destructively or coated in Au for SEM analyses.

### (b) Imaging and scanning electron microscopy

Specimens were photographed under alcohol in polarized and non-polarized light using a Canon EOS5 with macrolens. For each specimen, textural and compositional data on anatomical characters were collected using a Hitachi S-3600N environmental scanning electron microscope with Oxford INCA 350 EDX system and a FEI Quanta 650 FEG SEM. Partial pressure was 20 Pa, working distance was between 9 and 12 mm, with an operating voltage of 15 kV. Specimens were uncoated.

### (c) Time of flight secondary ion mass spectroscopy

*Mayomyzon* specimen LEIUG123268 (figure 3) was rinsed with pure ethanol and then analysed by TOF-SIMS (TOF.SIMS 5, ION-TOF GmbH, Germany 2010). The analysis beam, consisting of 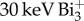 18 ns pulses (high current bunched mode, 10 kHz rate and 0.9 pA measured sample current), was typically raster scanned at 256 × 256 pixels over 500 × 500 µm areas. To reduce the intrinsic adventitious contamination induced by specimen storage in air the locations of interest were sputtered for 5 min with a Cs^+^ beam (2 keV ion energy, 90 nA measured sample current) and data were acquired before and after. Negative ions were collected over a mass range of *m/z* = 0–880 atomic mass units, with an average mass resolution (*m*/*δm*) about 2000 (figure 3*c*). The area of interest (figure 3*b*) included the dorsal part of the eye as well as the adjacent matrix. The secondary ion maps generated for specific fragments previously assigned to eumelanin [12] indicate the same localization of these fragments with the fossil eye (electronic supplementary material, figure S4). Other secondary ions (e.g. FeO^−^, ^57^FeO^−^, 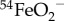, and Si_4_O_2_H^−^) were mapped in both the sediment and the eye, or predominantly in the sediment (figure 3*d*). On the same specimen, an area of the dorsal trunk dark stripe was similarly analysed with Bi+ primary ions (not shown). Multivariate statistical comparison of spectra (principal components analysis) followed the methods of [8,15]. The use of elevated temperature pressure experiments to biological organic material is a well-established approach to understand thermal maturation and the effects of ‘fossilization’ processes [39]. The artificial maturation of samples used here (see the electronic supplementary material, figure S5) follows the protocol described in [15].

## Supplementary Material

Details on the anatomy of Mayomyzon

## Data accessibility

The data we used in this publication can be accessed from Dryad at doi:10.5061/dryad.6m3n9.
