# Supplementary material for: Pigmented anatomy in Carboniferous cyclostomes and the evolution of the vertebrate eye
Source: Proc Biol Sci. 2016 Aug 17;283(1836):20161151. doi: 10.1098/rspb.2016.1151 (PMC5013770; doi:10.1098/rspb.2016.1151)
Supplement: Details on the anatomy of Mayomyzon [file rspb20161151supp1.docx]

**Supporting Electronic Supplementary Material**

**for**

**Pigmented anatomy in Carboniferous cyclostomes, and the evolution of the vertebrate eye**

**Gabbott, S. E., Donoghue, P. C. J., Sansom, R. S. Vinther, J., Dolocan, A. & Purnell, M. A.**

***1.* Details on the anatomy of *Mayomyzon***

Scanning Electron Microscopy and EDX analyses have resolved characters in specimens of *Mayomyzon* including an oral disc, an eye lens, otic capsule with statoliths, which have not been recognised, figured and/or fully described previously. This new anatomy was included in the phylogenetic analyses.

**Oral disc.** Three specimens (ROMV56800, PF5687 and ROM 56787) preserve at the anterior-most extremity, and slightly ventrally a clear semi-circular structure with radiating lines which is partly buried within the sediment. This structure is up to 1200 µm in diameter and is moldic (Supplementary Figure 1). This is best interpreted as an oral disc.

**Eye lens.** Specimen PF5687 is preserved in lateral aspect with a slight offset such that the two eyes appear as overlapping circles (Supplementary Figure 1). Both are composed of carbon and comprise melanosomes. Each eye contains a smaller circle (380 µm by 500 µm), centrally positioned, defined topographically and as an area of relatively low carbon abundance with respect to the surrounding eye when mapped using EDX. These smaller circles have an identical offset as the eyes (larger dark circles) and we interpret them as eye lenses. Similar features within the eye can be seen in other specimens (ROM56787b; ROM56806 and PF5539).

**Otic capsule and statoliths**. Two laterally-collapsed specimens of *Mayomyzon* (ROMV56800; PF5687) preserve two closely overlapping ring-shaped structures on both part and counterpart positioned immediately posterior to the eyes. In normal light they appear distinctly golden. Scanning Electron Microscopy of specimen ROMV56800 (Supplementary Figure 2) shows this structure to be approximately 1750 µm in diameter and it is delineated by a mass of subhedral cubic, octahedral and pyritohedral pyrite crystals (typically 9 µm across). The margin between the edge of the ring and the centre is indistinct. In the centre of the ring occur tens of smooth spherical structures (4.0 µm – 9.0 µm in diameter) which are composed of calcium phosphate. Based on their morphology, topology and presence of apatitic spheres we interpret the ring-shaped structures as the paired otic capsules and the contained spheres as statoliths. Extant lamprey statoliths are apatitic. The interpretation of the otic capsules in *Mayomyzon* allows us to be more confident of the interpretation of the paired, carbonaceous dark structures as eyes and not otic capsules.

**Fin radials.** Specimen (ROMV56800) preserves clear fin radials in the dorsal portion of the caudal fin (Supplementary Figure 2).

**Dark axial line**. The axial line in *Mayomyzon* dominantly comprises a carbon film but some melanosomes are preserved in both laterally (PF5687, LEIUG 123268, ROM56787, ROM56800, ROM56788, ROM56806, ROM56828) and dorso-ventrally (PF10788) collapsed specimens. The axial line has been interpreted as the digestive tract[3, 4]. A further possibility for specimens which are laterally collapsed is that the dark axial line is the expression of the lateral line, the neuromasts of which are known to be highly pigmented in many extant lamprey taxa[5]. Another possibility is that the melanosomes occur on this feature where the broadly-spaced bars of melanin intersect with the axial line.

**
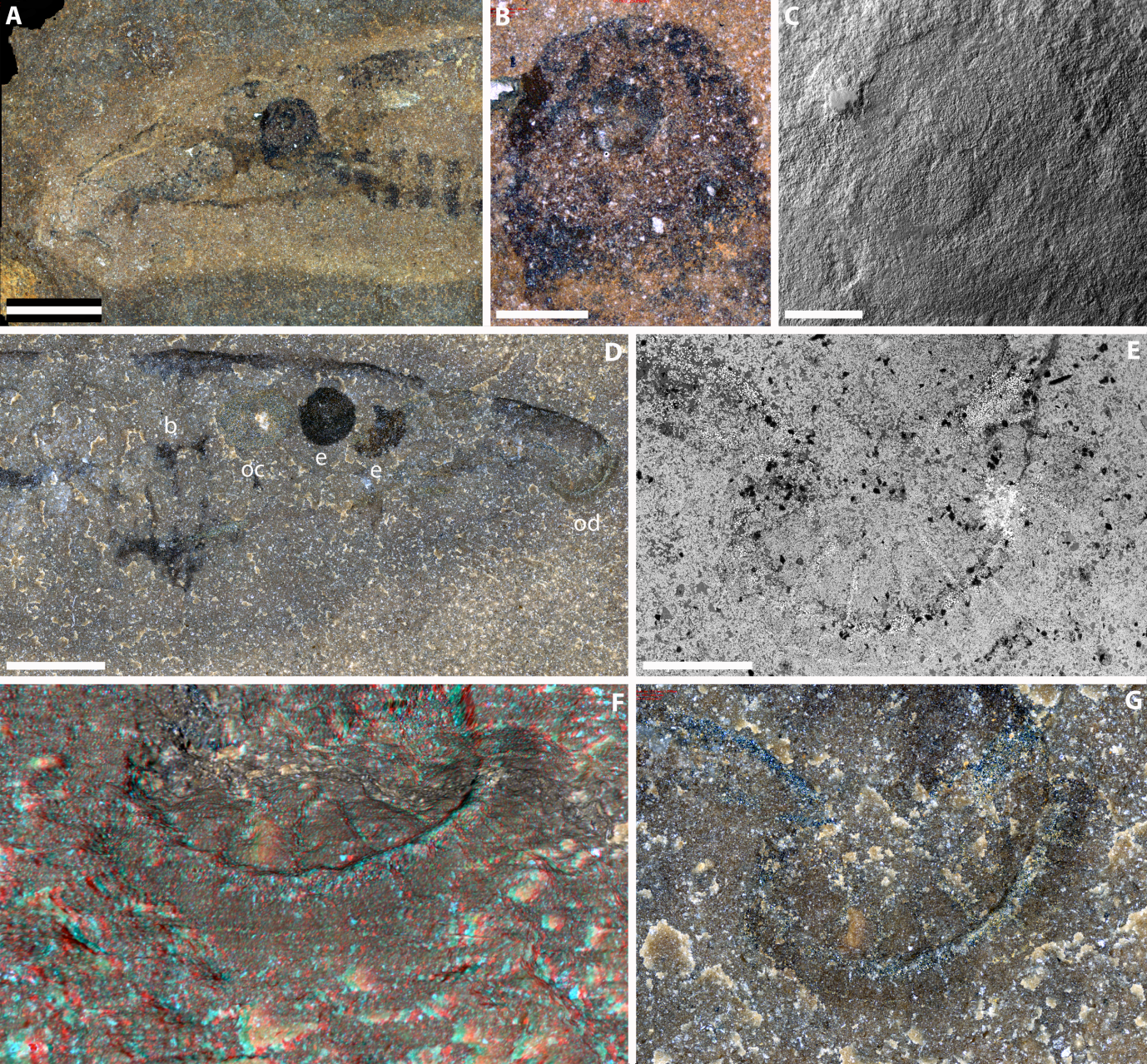
**

**Figure S1, Related to Figures 1 & 2**

**Details of *Mayomyzon* *pieckoensis* anatomy: A-C specimen PF5687, D-G specimen ROMV56800b.**

(A) slight oblique lateral collapse is demonstrated by marginally offset eyes (the near-side eye is positioned slightly dorsal). Within the dorsal-most eye a small dark circular disc with relief is interpreted as the lens. Scale bar = 2 mm.

(B) optical image of eye with centrally positioned lens. Scale bar = 500 µm.

(C**)** back-scatter electron image of the same area showing in relief two overlapping offset eyes, both with centrally-positioned small discs representing the lens from each eye. Scale bar = 500 µm.

(D) anterior of ROMV56800b showing two slightly offset eyes, an otic capsule and oral disc. e, eye; oc, otic capsule, b, branchial structure; od, oral disc. Scale bar = 200 µm.

(E, F, G) images of the oral disc in ROMV56800b. (E) back-scatter electron image. The bright minerals concentrated in the negative relief between the plates in the oral disc are pyrite. Scale bar = 500 µm. (F) anaglyph image and (G) optical image of the same view as E.

**
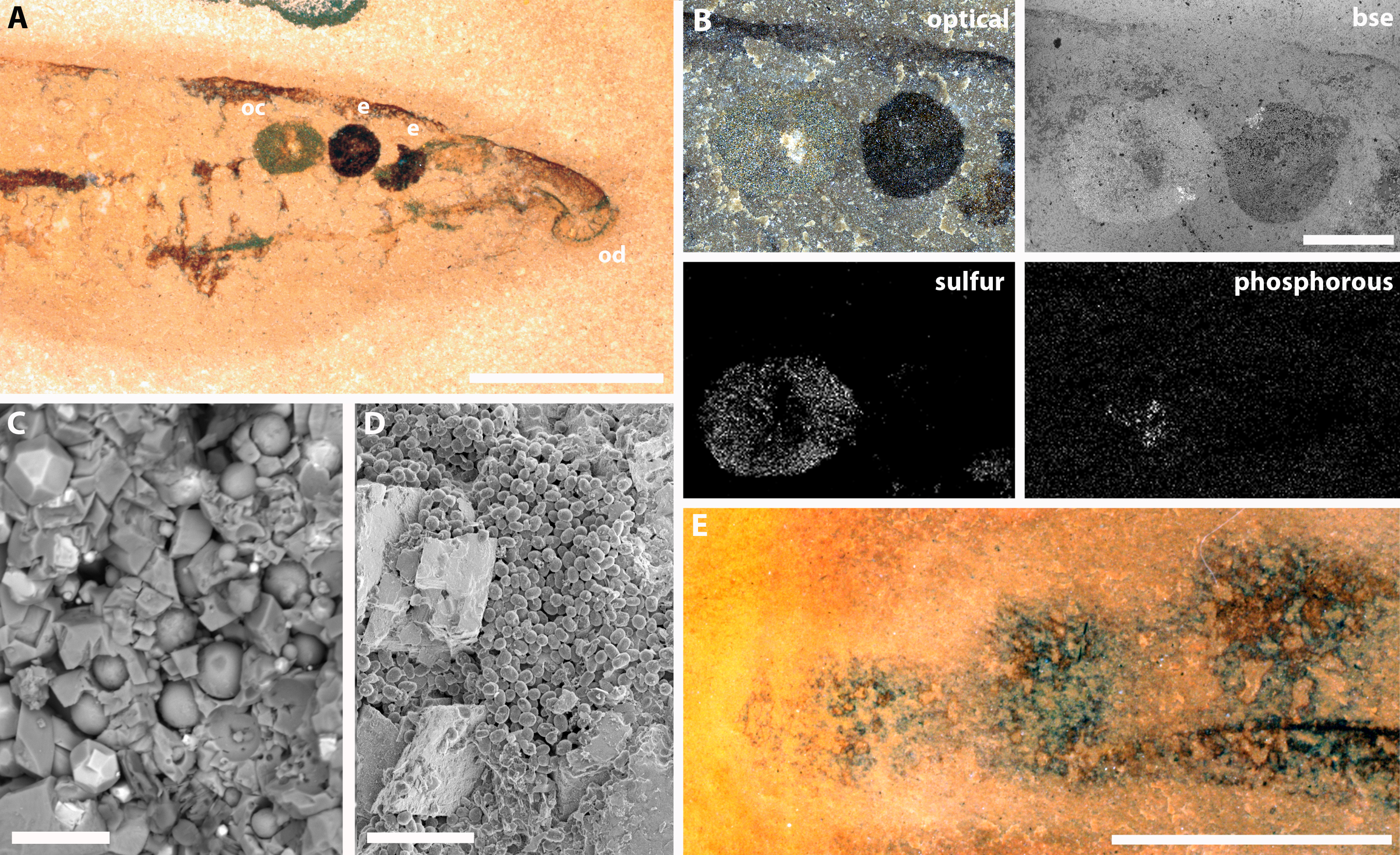
**

**Figure S2, Related to Figures 1 & 2**

**Details of *Mayomyzon* *pieckoensis* anatomy ROM 56800a,b.**

(A) anterior showing two slightly offset eyes, an otic capsule and oral disc. *e, eye; oc, otic capsule; od, oral disc.* Scale bar = 5 mm.

(B) close-up images of the eye and otic capsule in optical and back-scatter electron conditions and elemental maps of the same area for sulphur and phosphorous. The otic capsule comprises pyrite (Fe and S are relatively high with respect to the fossil and matrix) and the contained statoliths are composed of apatite (Ca and P are relatively high with respect to the fossil and matrix). Scale bar for all images = 1 mm.

(C) image of the centre of the otic capsule showing euhedral pyrite crystals and smooth spheres of apatite which are interpreted as statoliths. Scale bar = 5 µm.

(D) melanosomes comprising dorsal dark coloured bars ‘stripes’. The large diamond-shaped minerals on the left hand side of the image are siderite. Scale bar = 5 µm.

(E) the caudal fin displaying finely-spaced fin radials. Scale bar = 5 mm.

**

**

**Figure S3, Related to Figure 1.**

**Composition of the eye of *Mayomyzon* *pieckoensis*: specimen PF5687.**

**(A)** back-scatter electron image of the eye, anterior is to the left; an optical image of the same view is shown Supplementary Figure 1B.

**(B)** EDX map for carbon of the same area. No other element corresponds with the position of the eye. All scale bars = 1 mm.

**
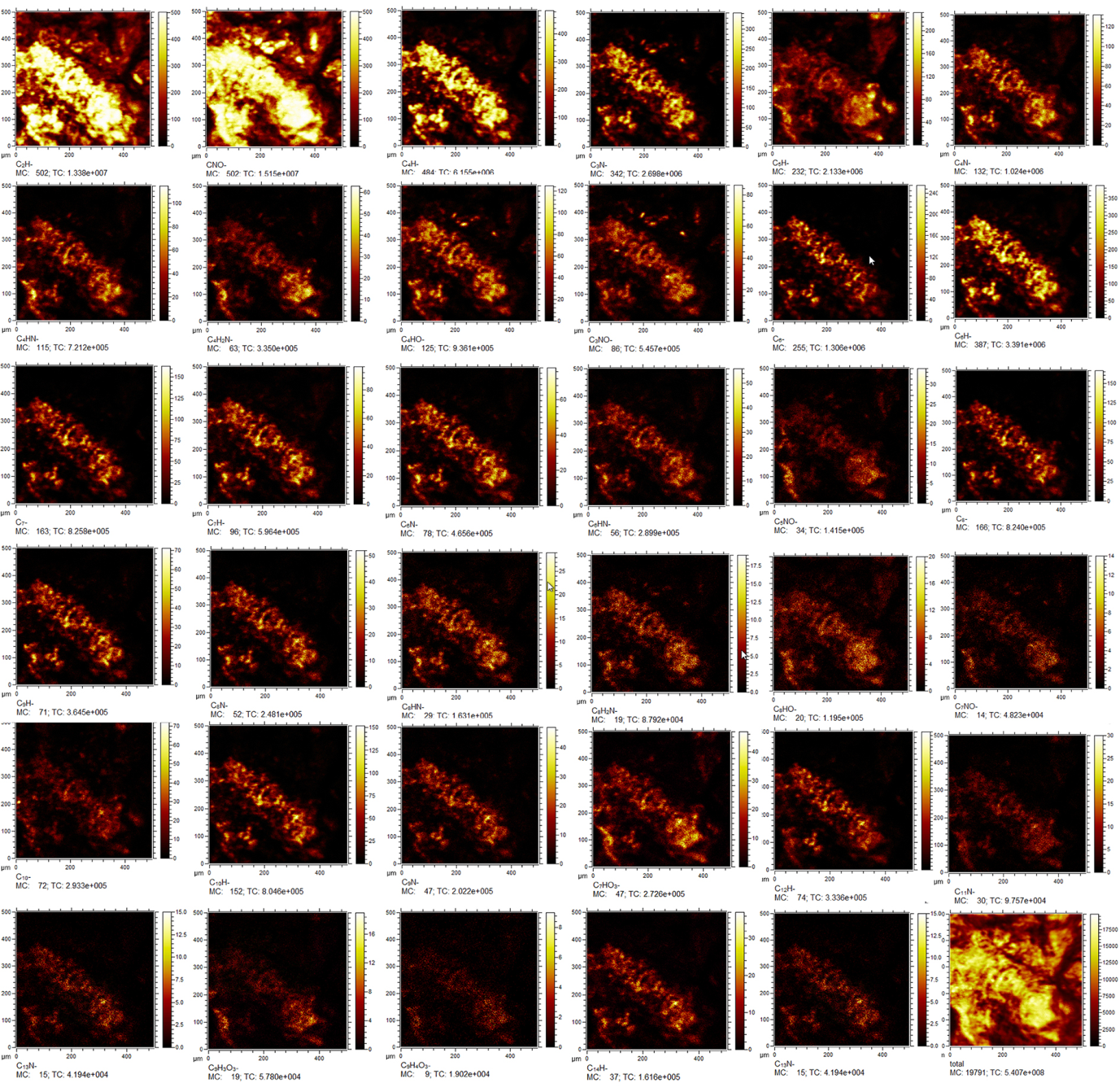
**

**Figure S4, Related to Figure 3**

**Negative secondary ion maps from a host of organic fragments previously recognized in eumelanin[1, 2]; same field of view as Figure 3.**

**
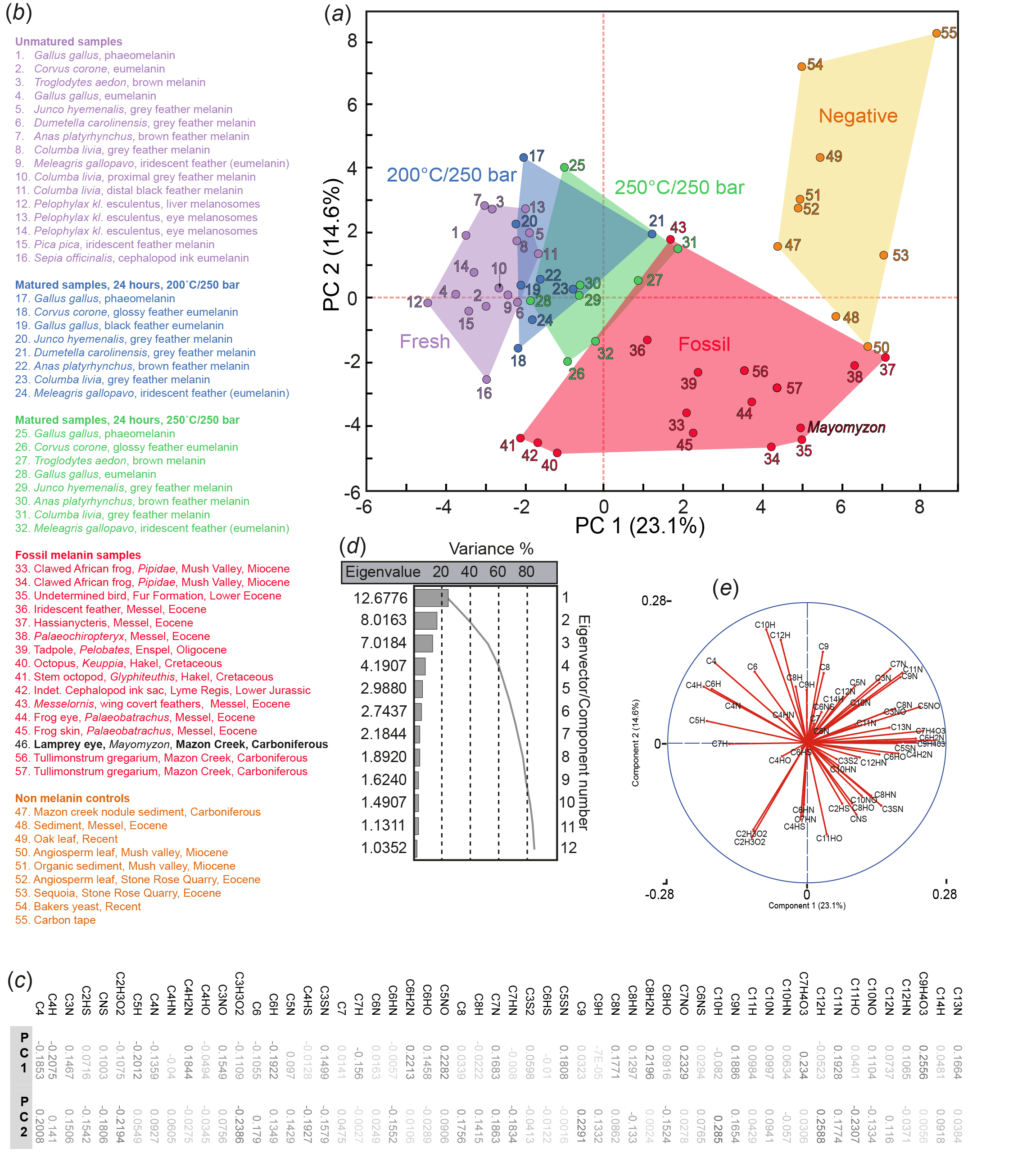
**

**Figure S5, related to Figure 3**

1. **PCA analysis of the area counts from 55 secondary ion peaks associated with melanins from fresh, artificially matured (24h at 200°C/250 bar 250°C/250 bar) and fossil melanin samples, as well as melanin-negative samples, from Colleary et al. 2015. *Mayomyzon* plots among other melanin-bearing samples and near other samples of fossil RPE (e.g. *Tullymonstrum*, a vertebrate from the Mazon Creek and a frog eye from Messel). Note that artificially matured melanins plot closer to fossil samples suggesting diagenetic alteration of fossil melanin Colleary et al. 2015. (b) list of samples in the PCA plot. (c) Eigenvector values for principal components 1 and 2, with intensity of shading for eigenvector values proportional to their loading on the principal component axes. (d) Eigenvector values for the first 12 principal components and the percentage of variation accounted for by each. (e) loading plot showing loading and orientation of eigenvectors relative to PC axes. Fragments such as C_n_NH-, C_n_NO-, C_n_NS-, C_n_SH- and C_n_OH- are mostly responsible for the separation of fossil melanin in the PCA space, whereas fragments such as C_n_- and C_n_H- separate the fresh melanin. This indicates both the chemical degradation (loss of carbon, nitrogen, sulphur and water) and structural degradation (weaker molecular bonding) of melanin during the fossilization process (Colleary et al. 2015; Clements et al. 2016). PCA plot modified from Clements et al. 2016.**

**Supplementary figure S6**

**Negative TOF SIMS spectra from the preserved eye in *Mayomyzon* LEIUG 123268 in comparison to the adjacent sediment and to a fossil melanin reference (Jurassic cephalopod ink). Red crosses indicate secondary ions, characteristic of melanin and used in PCA analysis (Colleary et al. 2015). Spectra from the fossil eye are distinct from spectra of the adjacent matrix, but contain several inorganic ions (Fig. 3). This makes visual comparison to other melanin samples difficult. In order to view the distinct contribution from the melanin characteristic secondary ions in the fossil eye, compared to the sediment, the lower two spectra show these extracted from the eye and sediment. These ions are lowly expressed in the sediment and have different relative intensity. Intensity measured in number of secondary ion counts to the detector along the y-axis and M/Z in atomic mass units along x-axis.**

**2. Phylogenetics**

The assembled data matrix is a revision of another [6], but much revised in terms of character state scores; these amendments are recorded in relation to the character descriptions. In particular we reanalysed the anatomy of *Myxinikela* based on detailed observations (including SEM) of the holotype and considered it in light of data from taphonomy experiments (Sansom et al. 2011). Below we test the prior hypothesis that *Myxinikela* is a hagfish using statistical phylogenetic methods and find that it cannot be rejected. This is the most robust test of the affinity of *Myxinikela’s* affinity conducted – indeed, it is the most robust test employed to date to address the affinity of any early vertebrate.

The matrix was analysed using both parsimony and Bayesian phylogenetic inference. Parsimony analyses were conducted using both PAUP [7] and TNT [8] while Bayesian analyses were performed using MrBayes [9]. We explored the impact of implied weighting in TNT by subjecting the strict component consensus of the most parsimonious trees derived using a range of concavity constant (K) values including ∞ (equal weights) and 99 further K values drawn from a log normal distribution. The consensus network, was calculated using SplitsTree [10] V4.13.1. In all instances, analyses were rooted on Cephalochordata, following Delsuc et al. [11]. In both parsimony and Bayesian analyses, we conducted unconstrained tree searches, but also tree searches constrained by a backbone topology compatible with cyclostome monophyly – for which there is overwhelming molecular evidence [12-14]. By implication, hagfishes and lampreys have lost characters inherited from the ancestral crown vertebrate, a view corroborated by analyses of hagfish embryology that have revealed the cryptic presence of anatomical characters previously thought present only in lampreys and jawed vertebrates [15-21]. PAUP analyses were conducted using the ‘branch and bound’ search option. Each MrBayes analysis was run for 1 million generations, sampling every 1000 generations, and consisting of 1 cold and 3 incrementally heated chains, with a burnin of 25%. Replicate runs converged (effectively identical traces with PRSF of ~1.00 and standard deviation of split frequencies <0.02).

In attempting to establish the phylogenetic affinity of *Myxinikela*, we sought to establish not only the most parsimonious or likely topologies but, also, whether the prior hypothesis of a hagfish affinity could be rejected based on the dataset. The statistical tests we employed within the parsimony analyses were the Templeton Test (Templeton 1983), Kishino-Hasegawa Test (Kishino and Hasegawa 1989), and an approximation of the Shimodaira-Hasegawa Test (Shimodaira and Hasegawa 1999; Goldman et al. 2000); within the Bayesian analyses we attempted to discriminate statistically among competing phylogenetic hypotheses using Bayes Factors (Nylander et al. 2004). The Templeton and Kishino-Hasegawa tests are only applicable when the competing topologies are established *a priori*, which is appropriate here. To this end, we sought to discriminate among the competing hypotheses that *Myxinikela* is a stem-craniate, stem-vertebrate, stem-cyclostome, stem-lamprey, and stem-gnathostome. The samples of trees associated with these competing topologies were obtained using backbone/partial constraint trees compatible with these hypotheses. The Shimodaira-Hasegawa Test was developed as a one-tailed alternative to the two-tailed Templeton and Kishino-Hasegawa tests and, though it has not been implemented for phenotypic data, we employed its approximation, as suggested by Goldman et al. (2000; i.e. half of the *P* value).

***Parismony analysis*.** Unconstrained and unweighted analysis of the dataset using the random sequence addition heuristic search algorithm (10 replicates with 10 trees retained at each step) yielded 5226 trees at 161 steps (CI: 0.6894; RI: 0.7525; RC: 0.5188; Figure S7A) in which the phylogenetic relationships of *Myxinikela* and Myxinoidea are unclear in the strict consensus (although their sister relationships are supported in two thirds of trees in the majority rule consensus of the 5226 trees). Deletion experiments revealed that much of this uncertainty can be attributed to *Haikouichthys* and *Achanarella*; when these taxa are excluded, unweighted analysis yields 90 equally most parsimonious trees at 158 steps (CI: 0.6962; RI: 0.7551; RC: 0.5257; Figure S7B). *Myxinikela* and Myxinoidea are resolved as sister taxa in the strict consensus of these 90 trees.

Figure S7: Strict consensus of equally most parsimonious trees derived from unconstrained analysis of the complete dataset (A) and after deletion of *Haikouichthys* and *Achanarella*.

Parsimony analysis of the complete dataset enforcing a backbone constraint tree compatible with cyclostome monophyly yielded 1128 equally most parsimonious trees at 168 steps (CI: 0.6647; RI: 0.7228; RC: 0.4804; Fig. S8A). Deletion of *Haikouichthys* and *Achanarella* yielded 90 equally most parsimonious trees at 165 steps (CI: 0.6667; RI: 0.7194; RC: 0.4796; Fig. S8B).

Figure S8: Strict consensus of equally most parsimonious trees derived from cyclostome-monophyly constrained analysis of the complete dataset (A) and after deletion of *Haikouichthys* and *Achanarella*.

We conducted statistical tests on the subsampled dataset (excluding *Haikouichthys* and *Achanarella*). All topology tests under parsimony failed a test of statistical significance indicating that none can be discriminated based on the data, including cyclostome monophyly versus paraphyly and, within these constraints, the possibility that *Myxinikela* is a stem-craniate, -vertebrate, -cyclostome, -hagfish, -lamprey, and stem-gnathostome. That being so, we also cannot reject the prior hypothesis that *Myxinikela* is a total group hagfish.

*SpitsTree analysis. Myxinikela* consistently groups with Myxinioidea in all of the strict component consensus trees derived from the different k-states employed in the implied weighting methods of TNT, as is clear from the consensus networks derived from SplitsTree analysis of these trees (Figs S9, 10).

Figure S9: Consensus network at 0.0 threshold showing all of the relationships expressed in the strict component consensus trees derived from 100 different K values in an implied weighting parsimony analysis of the data in TNT, assuming cyclostome paraphyly. *Myxinikela* always resolves at the sister group to extant Myxinoidea.

Figure S10: Consensus network at 0.0 threshold showing all of the relationships expressed in the strict component consensus trees derived from 100 different K values in an implied weighting parsimony analysis of the data in TNT, assuming cyclostome monophyly. *Myxinikela* always resolves at the sister group to extant Myxinoidea.

The Bayesian phylogenetic analyses were conducted unconstrained, recovering cyclostome paraphyly, and with backbone constraint enforcing cyclostome monophyly. The BF_KR_[22] between these two topologies ranged 5.094-14.452, indicating weak to decisive support for cyclostome paraphyly, given the data. *Myxinikela* and Myxinoidea are consistently resolved as sister taxa, with strong support (87%) within the context of cyclostome monophyly, and weak support (59%) assuming cyclostome paraphyly.

Figure S11: Summary tree from Bayesian analysis of the dataset, assuming A: cyclostome paraphyly, and B: cyclostome monophyly. Clade credibilities reflect support for their respective nodes.

Statistical support is not high across the taxa and the analyses. However, *Myxinikela* and Myxinoidea are consistently resolved as each others sister lineages across all analyses, including those integrating phylogenetic uncertainty. Thus, we conclude that this is the best-supported hypothesis for the phylogenetic position of *Myxinikela* and, at the least, we have not been able to reject this prior hypothesis.

**Supplemental References**

1. Lindgren, J., Uvdal, P., Sjövall, P., Nilsson, D.E., Engdahl, A., Schultz, B.P., and Thiel, V. (2012). Molecular preservation of the pigment melanin in fossil melanosomes. Nat Commun 3, 824.

2. Lindgren, J., Sjövall, P., Carney, R.M., Uvdal, P., Gren, J.A., Dyke, G., Schultz, B.P., Shawkey, M.D., Barnes, K.R., and Polcyn, M.J. (2014). Skin pigmentation provides evidence of convergent melanism in extinct marine reptiles. Nature.

3 Bardack, D. & Zangerl, R. First fossil lamprey: a record from the Pennsylvanian of Illinois. *Science* **162**, 1265-1267 (1968).

4 Bardack, D. & Zangerl, R. Lampreys in the fossil record. *The biology of lampreys* **1**, 67-84 (1971).

5 Renaud, C. Lampreys of the world. An annotated and illustrated catalogue of lamprey species known to date. FAO species catalogue for fisheries purposes No. 5.–Rome: FAO. 109 p. *GA Shandikov* (2011).

6 Sansom, R. S., Freedman, K., Gabbott, S. E., Aldridge, R. J. & Purnell, M. A. Taphonomy and affinity of an enigmatic Silurian vertebrate, *Jamoytius kerwoodi* White. *Palaeontology* **53**, 1393-1409 (2010).

7 PAUP*: Phylogenetic Analysis Using Parsimony *and other methods. v. Version 4.0b10 (Sinauer Associates, Washington DC, 2002).

8 TNT (Tree analysis using New Technology) (Published by the authors, Tucumán, Argentina, 2000).

9 Ronquist, F. *et al.* MrBayes 3.2: efficient Bayesian phylogenetic inference and model choice across a large model space. *Systematic Biology* **61**, 539-542, doi:10.1093/sysbio/sys029 (2012).

10 Huson, D. H. & Bryant, D. Application of phylogenetic networks in evolutionary studies. *Molecular Biology and Evolution* **23**, 254-267 (2006).

11 Delsuc, F., Brinkmann, H., Chourrout, D. & Philippe, H. Tunicates and not cephalochordates are the closest living relatives of vertebrates. *Nature* **439**, 965-968 (2006).

12 Heimberg, A. M., Cowper-Sal lari, R., Sémon, M., Donoghue, P. C. J. & Peterson, K. J. microRNAs reveal the interrelationships of hagfish, lampreys, and gnathostomes and the nature of the ancestral vertebrate. *Proceedings of the National Academy of Sciences* **107**, 19379–19383, doi:http://www.pnas.org/lookup/suppl/doi:10.1073/pnas.1010350107 (2010).

13 Kuraku, S., Hoshiyama, D., Katoh, K., Suga, K. & Miyata, T. Monophyly of lampreys and hagfishes supported by nuclear DNA-coded genes. *Journal of Molecular Evolution* **49**, 729-735 (1999).

14 Smith, J. J. *et al.* Sequencing of the sea lamprey (Petromyzon marinus) genome provides insights into vertebrate evolution. *Nat Genet* **45**, 415-421, doi:http://www.nature.com/ng/journal/v45/n4/abs/ng.2568.html - supplementary-information (2013).

15 Ota, K. G., Kuraku, S. & Kuratani, S. Hagfish embryology with reference to the evolution of the neural crest. *Nature* **446**, 672-675 (2007).

16 Ota, K. G. & Kuratani, S. Cyclostome embryology and early evolutionary history of vertebrates. *Integr. Comp. Biol.* **47**, 329-337, doi:10.1093/icb/icm022 (2007).

17 Kuratani, S. & Ota, K. G. Hagfish (Cyclostomata, Vertebrata): Searching for the ancestral developmental plan of vertebrates. *BioEssays* **30**, 167-172 (2008).

18 Ota, K. G. & Kuratani, S. Developmental biology of hagfishes, with a report on newly obtained embryos of the Japanese inshore hagfish, *Eptatretus burgeri*. *Zoological Science* **25**, 999-1011 (2008).

19 Ota, K. G. & Kuratani, S. Phylogeny of early vertebrates based on evidence from developmental study of hagfish. *Integrative and Comparative Biology* **49**, e128 (2009).

20 Ota, K. G., Fujimoto, S., Oisi, Y. & Kuratani, S. Identification of vertebra-like elements and their possible differentiation from sclerotomes in the hagfish. *Nature communications* **2**, 373, doi:10.1038/ncomms1355 (2011).

21 Oisi, Y., Fujimoto, S., Ota, K. G. & Kuratani, S. On the peculiar morphology and development of the hypoglossal, glossopharyngeal and vagus nerves and hypobranchial muscles in the hagfish. *Zoological Letters* **1**, doi:10.1186/s40851-014-0005-9 (2015).

22 Kass, R. E. & Raftery, A. E. Bayes factors. *Journal of the American Statistical Association* **90**, 773-795 (1995).
